# Metagenomic Analysis of Saliva Reveals Disease-Associated Microbiotas in Patients With Periodontitis and Crohn’s Disease-Associated Periodontitis

**DOI:** 10.3389/fcimb.2021.719411

**Published:** 2021-09-27

**Authors:** Boyang Sun, Bingyao Liu, Xiaojiao Gao, Kai Xing, Li Xie, Ting Guo

**Affiliations:** ^1^ Nanjing Stomatological Hospital, Medical School of Nanjing University, Nanjing, China; ^2^ Jinling Hospital, Department of Stomatology, Nanjing Medical University, Nanjing, China; ^3^ Jinling Hospital, Department of Clinical Laboratory, Medical School of Nanjing University, Nanjing, China; ^4^ Jinling Hospital, Medical School of Nanjing University, Nanjing, China

**Keywords:** Crohn’s disease, periodontitis, salivary microbiota, metagenome, functional pathways, antibiotic resistance genes

## Abstract

Patients with Crohn’s disease frequently develop oral health problems and show a higher prevalence of oral manifestations, such as dental caries and periodontitis, than healthy individuals do. In this study, a metagenomic analysis was carried out to characterize the salivary microbiota in patients with either periodontitis or Crohn’s disease-associated periodontitis. Saliva samples were collected from six patients with both Crohn’s disease and periodontitis (Cm group), six patients with periodontitis alone (Pm group), and six healthy individuals (Hm group). Genomic DNA was collected from these samples for high-throughput Illumina HiSeq metagenomic sequencing. The composition of the bacterial communities and their metabolic pathways and gene functions were characterized and compared among the three study groups. The salivary microbial communities were significantly different among the three groups, with Firmicutes, Actinobacteria, and Bacteroidetes showing the most significant differences. The Cm and Pm groups had higher abundances of *Bacteroides fragilis*, *Prevotella baroniae*, *Prevotella enoeca*, and *Prevotella dentasini* than the Hm group. The Cm and Pm groups also showed differences in their salivary microbial communities, in that the Cm group had relatively high abundances of Firmicutes and Proteobacteria, whereas the Pm group had relatively high abundances of Actinobacteria, Bacteroidetes, and Fusobacteria. In total, 34 Pm-associated (e.g., Fusobacteria and *Corynebacterium matruchotii*), 18 Cm-associated (e.g., *Capnocytophaga* and *Streptococcus oralis*), and 18 Hm-associated (e.g., *Streptococcus* and Bacillales) predominant microbial species were identified. Most genes were involved in carbohydrate and amino acid metabolism, with those of the Cm and Pm groups showing more similarity to one another but significant differences from those of the Hm group. Most of the antibiotic resistance genes were found in the Pm group. In conclusion, the salivary microbial community structure and abundance were distinct among patients with Crohn’s disease-associated periodontitis, patients with periodontitis, and healthy individuals. Further studies are needed to evaluate the potential value of these microbiota and microbiome differences in the clinical diagnosis and treatment of oral diseases.

## Introduction

Crohn’s disease (CD), which comprises a major group of chronic recurrent inflammatory bowel disorders that are characterized by the diffuse inflammation of the intestinal mucosa, can affect any part of the digestive tract (Mak et al., 2020). Therefore, as part of the digestive tract, the oral cavity is frequently affected in patients with CD (Vavricka et al., 2013). Oral manifestations, such as dental caries and periodontitis, have been reported to occur at a higher rate in patients with inflammatory bowel disease than in healthy individuals (Koutsochristou et al., 2015; Poyato-Borrego et al., 2020).

The human body is inhabited by a large number of microbes that form complex ecosystems, with commensal microorganisms contributing to the health of the host by maintaining homeostasis, resisting pathogens, and modulating the immune system (Lloyd-Price et al., 2016). The human oral microbial community, which forms a crucial portion of the human microbiota, is a collection of all the microorganisms inhabiting the oral cavity and also represents an important portion thereof. The oral microbiota generally exists in the form of a biofilm and plays important roles in protecting the oral cavity against colonization by exogenous pathogenic bacteria, maintaining oral homeostasis, and preventing disease development (Arweiler and Netuschil, 2016; Zhang et al., 2018). The oral microbiome, which is the collection of genomes from all microorganisms in this environment, is closely related to oral tumors, diabetes, rheumatoid arthritis, cardiovascular disease, and other systemic diseases and has been considered potential biomarkers of human diseases (Jia et al., 2018; Graves et al., 2019). Periodontitis is associated with changes in the oral microbial community, being initiated by dysbiotic ecological alterations in the microbiome in response to gingival inflammation (Cui et al., 2019; Kleinstein et al., 2020). Studies have demonstrated that salivary microbes can be used to distinguish dental caries from periodontitis at the preclinical stages (Belstrøm et al., 2017). The oral cavity is the gateway through which microorganisms enter the digestive tract. On a daily basis, the average adult produces more than 1,000 ml of saliva, which contains a large number of oral bacteria, almost all of which enter the gastrointestinal tract (Carpenter, 2013; Pedersen et al., 2018). Therefore, through the human actions of eating and swallowing, oral microorganisms have a high opportunity of entering and colonizing the gastrointestinal tract and interfering with intestinal homeostasis.

CD and periodontitis, which are chronic inflammatory diseases mediated by the interaction between the host’s immune inflammatory response and the bacterial flora, both have relatively similar pathophysiological mechanisms and are influenced by genetic and environmental factors (Hong et al., 2016; Zhang et al., 2020). Oral and intestinal microorganisms interact structurally and functionally and play a joint role in disease processes (Ledder et al., 2008; Zhang et al., 2015). On the one hand, local microorganisms and their metabolites as well as the inflammatory cytokines that stimulate the host are important aggravating factors for systemic diseases (Abusleme et al., 2013; Hajishengallis, 2015). On the other hand, systemic factors also modify the host’s response to local factors, thereby affecting the course of periodontitis (Abusleme et al., 2013; Hajishengallis, 2015). Recent studies have shown that the diversity of the salivary microbiota and microbiome decreases in the active phase of CD in contrast to the diversity in the remission phase or in healthy individuals (Weingarden and Vaughn, 2017; Zhang et al., 2020).

Currently, knowledge about the correlation between periodontitis and CD in terms of the characteristics of the salivary microbiota is limited. Herein, we propose that dysbiosis of the salivary microbiota in CD is associated with periodontitis. Therefore, the objective of the current study was to explore the salivary microbiotas of patients with either periodontitis alone or CD-associated periodontitis. The findings of this study may be of clinical benefit for the diagnosis, treatment, and prognosis of gum disease.

## Materials and Methods

### Study Cohort Selection and Clinical Data Collection

From April 2018 to June 2019, 18 individuals were enrolled in the study at the General Hospital of Eastern Theater Command, Nanjing, China; that is, six patients diagnosed with both CD and periodontitis (Cm group), six patients with periodontitis alone (Pm group), and six healthy individuals (Hm group). All patients provided written informed consent before participation in the study, which was approved by the Ethics Committee of the Eastern Theater Command General Hospital (2019NZGKJ-077). All study procedures were performed in accordance with the Declaration of Helsinki.

CD was diagnosed on the basis of the Lennard–Jones criteria (Gomollón et al., 2017). All patients underwent a routine stool examination and colonoscopy, and a mucosal biopsy was confirmed pathologically. Periodontitis was clinically diagnosed according to a consensus report of Workgroup 2 of the 2017 World Workshop on the Classification of Periodontal and Peri-Implant Diseases and Conditions (Papapanou et al., 2018). The inclusion criteria for this study were as follows: (1) participants in the three groups were matched in terms of gender and age; (2) none of the participants had received any treatment for periodontitis or CD within the last six months; and (3) none of the participants had been treated with antibiotics for two weeks prior to the study. The exclusion criteria were as follows: (1) a history of antibiotic drug consumption within the past two weeks; (2) treatment for periodontitis or CD received within six months; (3) comorbidity of systemic diseases (cancer, diabetes mellitus, infectious disease, etc.); (4) serious psychological illnesses; and (5) a communication disorder or an inability to cooperate with the physician for the oral examination and sampling. For the Pm group, patients with CD or other intestinal inflammatory diseases were excluded. The healthy individuals were negative for CD and were periodontally healthy with a decayed, missing, and filled teeth (DMFT) score of less than or equal to 2.

Clinical and demographic characteristics were recorded for all the selected participants. The sex, age, and DMFT, periodontitis probing depth (PPD), bleeding on probing (BOP), and clinical attachment loss (CAL) scores of the individuals are summarized in **Table 1**.

**Table 1:** Clinical and demographic characteristics of the study participants.

Group	Gender	Age	DMFT	BOP (%)	PPD (mm)	CAL (mm)
Cm (n = 6)	3F, 3M	33.6	4.6 ± 2.3^a^ ^b^	44.5 ± 18.7^a^	4.3 ± 1.5^a^	3.8 ± 1.1^a^
Pm (n = 6)	3F, 3M	35.1	2.3 ± 1.7	42.8 ± 23.7^a^	4.0 ± 2.1^a^	4.5 ± 0.7^a^
Hm (n = 6)	3F, 3M	33.7	1.5 ± 1.5	4.2 ± 5.5	1.3 ± 1.1	0.3 ± 0.2

F, female; M, male; DMFT, decayed, missing, and filled teeth; BOP, bleeding on probing; PPD, periodontitis probing depth; CAL, clinical attachment loss; Cm, patients with Crohn’s disease and periodontitis; Pm, patients with periodontitis alone; Hm, healthy indiciduals.

a p < 0.05, compared with Hm.

b p < 0.05, compared with Pm.

### Saliva Collection

Saliva collection was performed according to the method described by Acharya et al. (2016). In brief, unstimulated whole saliva samples were collected from 9 a.m.–11 a.m. All participants were requested to refrain from drinking, smoking, eating, and oral hygiene activities (including rinsing with mouthwashes) for at least 1 h before saliva collection. Participants were allowed to pool the saliva in the mouth and then expectorate into a sterile 5 ml collection tube. The samples were placed on ice, and a protease inhibitor cocktail was added at a ratio of 100 μl per ml of saliva. The mixtures were then centrifuged at 6,000 × *g* for 5 min at 4°C, and the supernatants were collected and immediately frozen at –80°C until use.

### DNA Extraction and Sequencing

After the saliva supernatants had been thawed on ice, DNA was extracted from the samples using the QIAmp DNA Microbiome Kit (Qiagen, Valencia, CA, USA) and DNA Clean & Concentrator (ZYMO RESEARCH, Irvine, CA, USA) according to the respective manufacturer protocols. The DNA quantity was determined using a NanoDrop ND-1000 spectrophotometer and a Qubit fluorometer. Library construction was carried out using the NEBNext Ultra DNA Library Prep Kit for Illumina (NEB, Ipswich, MA, USA) according to the manufacturer’s protocol, and the DNA was sequenced on an Illumina HiSeq platform, generating paired-end reads. The raw sequencing data were deposited in the National Center for Biotechnology Information (NCBI) Sequence ReadArchive (SRA) database under accession number PRJNA741688.

### Data Quality Control and Metagenome Assembly

The raw sequencing data were first filtered to obtain clean data, following which read pairs shorter than 40 bp were filtered. Adapter sequence trimming from the 3′ end of the reads was then performed, with a quality threshold of 15 bp. To ensure that there was no potential host DNA contamination in the samples, reads were aligned to the human genome using Bowtie 2.2.4 (Langmead et al., 2009) for removal of those of host origin (parameters: –end-to-end, –sensitive, -I 200, and -X 400). Subsequently, the aligned reads were discarded, and the acquired clean dataset of high-quality reads was used for the downstream analysis. The high-quality reads were assembled into continuous sequences within scaffolds (scaftigs) using SOAP denovo, a short-read assembly method (parameters: -d 1, -M 3, -R, -u, and –F; K-mer = 55). The clean data from each sample were mapped against the scaftigs using Bowtie 2. The unmapped reads were continually assembled using a K-mer size of 55. Assembled sequences of less than 500 bp were discarded from the dataset.

### Gene Prediction

The scaftigs (≥500 bp) were used for open reading frame (ORF) prediction, which was performed with MetaGeneMark using the default parameters (Zhu et al., 2010; Karlsson et al., 2012; Qin et al., 2014). Predicted ORFs of less than 100 nt were discarded (Qin et al., 2010; Nielsen et al., 2014). Non-redundant gene catalogs were obtained using CD-HIT (parameters: -c 0.95, -G 0, -aS 0.9, -g 1, -d 0) (Li and Godzik, 2006; Fu et al., 2012) and clustered (95%; coverage 90%) to obtain the representative sequences (Li et al., 2014; Qin et al., 2014). Then, clean data from each sample were aligned with the gene catalog using Bowtie 2 (parameters: –end-to-end, –sensitive, -I 200, and -X 400) (Qin et al., 2010; Li et al., 2014). Genes of less than or equal to two reads were discarded. The obtained gene catalogs (unigenes) were then used for the taxonomic analysis.

### Taxonomic Analysis

The unigenes were aligned to sequences on the National Center for Biotechnology Information Non-Redundant (NR) database (v.2018.01; parameters: blastp, evalue ≤ 1e-5) (Karlsson et al., 2013), using DIAMOND software (Buchfink et al., 2015). Sequences with an e-value of less than or equal to the minimum e-value × 10 were reserved for subsequent analysis. Taxonomic binning was performed using the lowest common ancestor algorithm in MEGAN software (Huson et al., 2011). The top 10 species in terms of maximum relative abundance in each sample were selected, and histograms were plotted at different taxonomic levels. Principal component analysis (PCA) was used to display the differences in a two-dimensional coordinate chart (Rao, 1964). Non-metric multidimensional scaling (NMDS) analysis was applied to reflect the inter- or intra-group differences on the basis of the species information (Legendre and Legendre, 1998). The non-parametric test analysis of similarities (ANOSIM) was used to compare the differences among different groups at the phylum level. Metastats was used to compute *p*- and *q-*values for screening species with significant differences (White et al., 2009). The linear discriminant analysis (LDA) effect side (LEfSe) tool was used to screen for microbes that differed significantly among the three study groups (Segata et al., 2011).

### Functional Database Annotations

The unigenes were aligned to the Kyoto Encyclopedia of Genes and Genomes (KEGG) database (parameters: blastp, evalue ≤ 1e-5). Sequences with one high-scoring segment pair of greater than 60 bits were retained for the downstream analysis, and the number of genes was counted. The relative abundances of the annotated pathways were analyzed at three levels of the KEGG pathway maps and were represented by a heatmap or histograms. Metastats was used to analyze functional differences among the groups (White et al., 2009).

### Antibiotic Resistance Ontology Gene Analysis

Antibiotic resistance genes are ubiquitous in microorganisms in the human intestines and other environments. Antibiotic abuse results in irreversible changes to the microflora of the environment and human body, which are harmful to the ecosystem and human health. Hence, increasing attention has been paid to antibiotic resistance genes (Martínez et al., 2015). The unigenes were aligned to the Comprehensive Antibiotic Resistance Database using Resistance Gene Identifier software (parameters: blastp, evalue ≤ 1e-30). The relative abundances of the antibiotic resistance ontology (ARO) and resistance genes were analyzed according to the results.

### Statistical Analysis

IBM SPSS Statistics Desktop (SPSS, Chicago, IL, USA) was used for all data collection and statistical analyses. The cardinality test was used to compare the count data. The Spearman rank correlation test was used for correlation analysis. Comparisons of the measurement data between two groups were performed using the Wilcoxon signed-rank test. Student’s *t*-test was used to compare the diversity indices. Differences with a *p*-value of less than 0.05 were considered statistically significant.

## Results

### Clinical Characteristics of the Study Cohort

Our study cohort comprised six patients with both CD and periodontitis (Cm group), six patients with periodontitis alone (Pm group), and six healthy individuals (Hm group). There were no significant differences among the individuals in the three study groups in terms of sex (*p* > 0.05) or age (*p* > 0.05) (**Table 1**). The patients in the Cm group had the highest DMFT scores (*p* < 0.05). The mean BOP, PPD, and CAL scores were significantly lower in the Hm group than in the Cm (*p* < 0.05) and Pm groups (*p* < 0.05). However, there were no significant differences in the BOP, PPD, and CAL scores between the Cm and Pm groups (*p* > 0.05).

### Distribution of Common Taxa in the Three Study Groups

**Figure 1:**
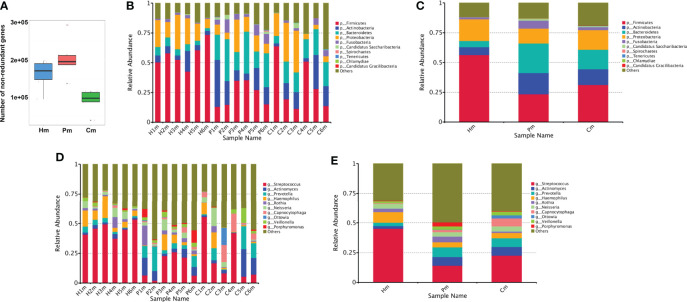
Number of non-redundant genes and taxonomic distribution in the oral microbiome of individuals in the Hm, Cm, and Pm groups. **(A)** Box plot indicating the number of non-redundant genes. Barplots of the relative abundances of different taxa at the phylum level among **(B)** all the samples and **(C)** different groups. Barplots of the relative abundances of different taxa at the genus level among **(D)** all the samples and **(E)** different groups. Hm, healthy individuals; Pm, patients with periodontitis alone; Cm, patients with Crohn’s disease and periodontitis.

In total, 193,970.59 Mbp of raw data was generated from the 18 saliva samples, with a mean of 10,776.14 Mbp of data per sample. After quality control, 193,820.26 Mbp of clean data was obtained, with an effective percentage of 99.92% (**Table S1**). After redundancy removal, 435,360 ORFs were obtained, with a total length of 284.96 Mbp and an average length of 654.54 bp. Of these, 200,361 complete genes were found, accounting for 46.02% of the total number of non-redundant genes (**Table S2**). The number of non-redundant genes in the Hm group was less than that in the Pm group and more than that in the Cm group (**Figure 1A**).

Of the 435,360 predicted genes, 373,894 (85.88%) were annotated to the NR database, of which 93.41% were annotated to the boundary level. The proportions for the phylum, class, order, family, genus, and species levels were 91.91%, 90.60%, 88.94%, 87.04%, 83.58%, and 54.13%, respectively. Overall, there were significant differences in the microbial composition among the three study groups. At the phylum level, samples from the Pm and Cm groups had a significantly lower abundance of Firmicutes and Proteobacteria than those from the Hm group. By contrast, samples from the Pm and Cm groups had a significantly higher abundance of Actinobacteria, Bacteroidetes, and Candidatus Saccharibacteria than those from the Hm group. Additionally, there were differences between the Pm and Cm groups, with the latter group having a relatively higher abundance of Firmicutes and Proteobacteria and the former group having a relatively higher abundance of Actinobacteria, Bacteroidetes, and Fusobacteria (**Figures 1B, C**). At the genus level, the Hm group had a higher abundance of *Streptococcus* and *Haemophilus* and a lower abundance of *Actinomyces*, *Prevotella*, *Capnocytophaga*, and *Veillonella* than did the other two groups. By contrast, the Pm and Cm groups had a relatively higher abundance of *Rothia* and *Porphyromonas*. The Cm group had a relatively higher abundance of *Ottowia* than did the Pm and Hm groups (**Figures 1D, E**).

**Figure 2:**
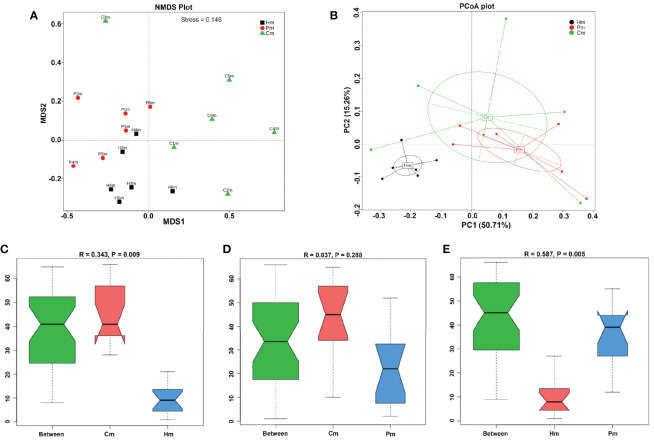
Comparison of the microbial compositions among the various study groups. **(A)** NMDS plots of the microbiotas in individuals in the Hm, Pm, and Cm groups; the distance between points indicates the degree of variation; a stress value < 0.2 indicates that the NMDS analysis is reliable. **(B)** PCA plots of the microbiotas in individuals in the three groups, based on Bray–Curtis distances. Samples with high similarity in their community composition tend to cluster together. **(C–E)** ANOSIM test of whether the difference among groups is significantly greater than the difference within groups; an R-value < 0 indicates that the intra-group difference is greater than the inter-group difference; *p* < 0.05 indicates the significance. Hm, healthy individuals; Pm, patients with periodontitis alone; Cm, patients with Crohn’s disease and periodontitis; NMDS, non-metric multidimensional scaling; PCA, principal components analysis; ANOSIM, analysis of similarities.

NMDS analysis was performed to verify the differences among the Cm, Hm, and Pm groups in terms of microbiota composition. Results indicated that the changes in the microbial composition could be inferred from the genetic data, since the stress value was 0.146 (i.e., <0.2), which corresponded closely to that of the PCA (**Figures 2A, B**). ANOSIM, a nonparametric test, was conducted to determine whether the differences among the groups were significantly greater than those within groups; that is, whether the grouping was meaningful. At the phylum level, all the R-values obtained were greater than 0, suggesting that the differences among the groups were greater than the differences within each group (**Figures 2C–E**). These results proved the reliability of this grouping.

### Identification of Species With Different Abundances in the Samples

**Figure 3:**
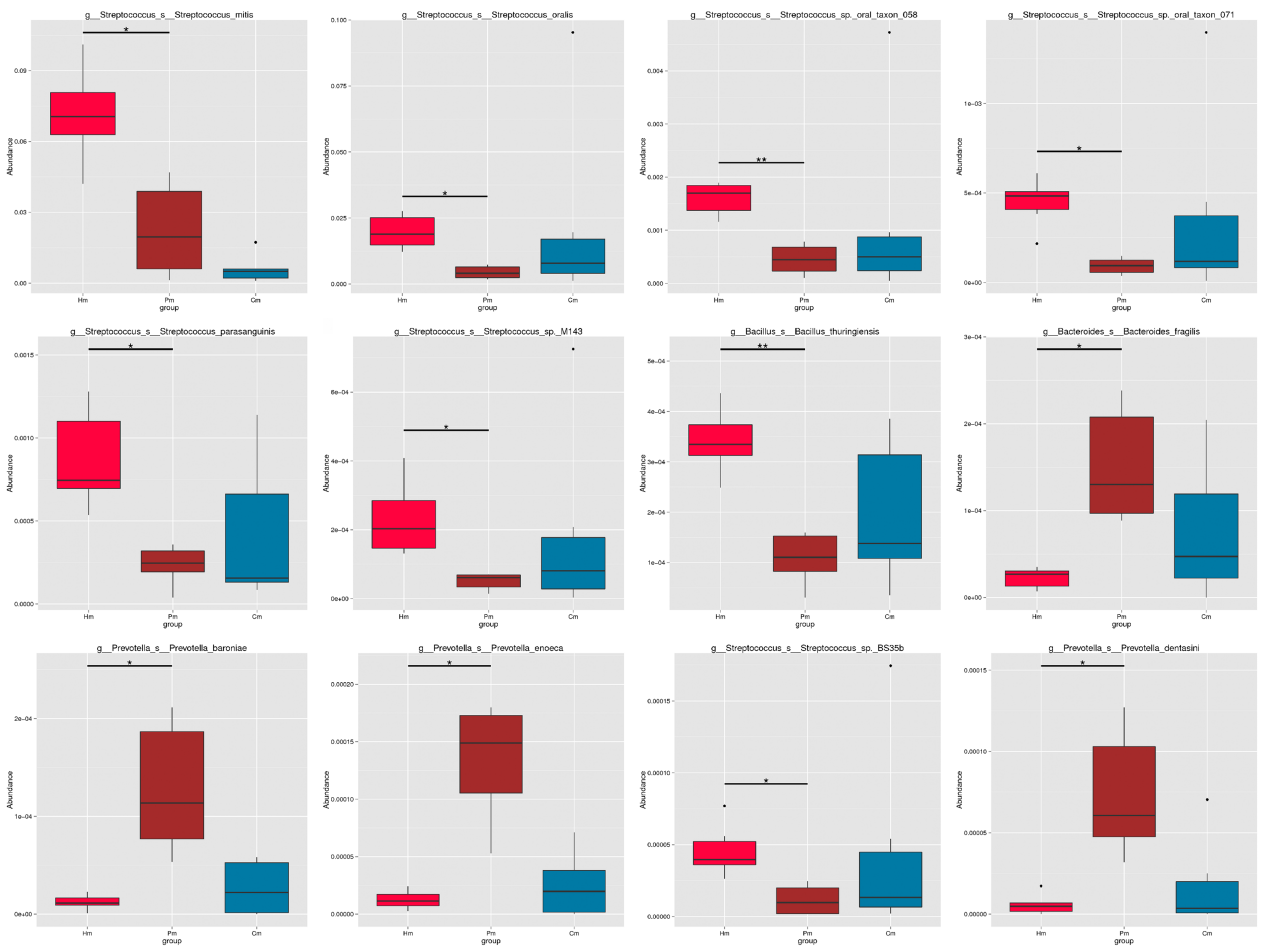
Differences in the abundance of 12 genera among the Hm, Pm, and Cm groups. Metastats was used to test the differences in the microbial composition between groups, with *q*-values computed for the multiple comparisons. *q-value < 0.05; **q-value < 0.01. Hm, healthy individuals; Pm, patients with periodontitis alone; Cm, patients with Crohn’s disease and periodontitis.

Metastats was used to investigate the significant differences among the Pm, Cm, and Hm groups at the species level. The Hm samples had a higher abundance of *Streptococcus mitis*, *Streptococcus oralis*, *Streptococcus* sp. oral taxon 058, *Streptococcus* sp. oral taxon 071, *Streptococcus parasanguinis*, *Streptococcus* sp. M143, *Bacillus thuringiensis*, and *Streptococcus* sp. BS35b than did the Pm and Cm samples. By contrast, the Hm group had a lower abundance of *Bacteroides fragilis*, *Prevotella baroniae*, *Prevotella enoeca*, and *Prevotella dentasini* than did the other two groups (**Figure 3**). The predominant microbial species in the three groups are shown in **Figure S1**, in which 34 Pm-associated (including *Fusobacteria*, *Corynebacterium matruchotii*, *Porphyromonas gingivalis*, Prevotellaceae, and *Alloprevotella tannerae*), 18 Cm-associated (e.g., *Capnocytophaga*, *Streptococcus oralis*, *Pseudopropionibacterium*, and Unclassified_Bacteria), and 18 Hm-associated (including *Streptococcus*, *Streptococcus mitis*, *Gemella haemolysans*, and Bacillales) microbial species were identified.

### Functional Characteristics of the Differentially Abundant Species in the Samples

**Figure 4:**
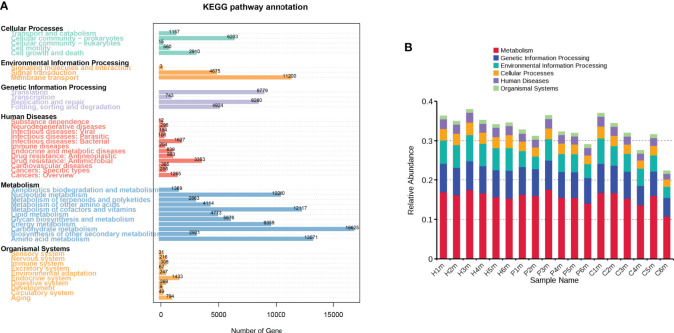
KEGG level 1 pathway annotation according to the genetic data of the oral microbiomes from individuals in the Hm, Cm, and Pm groups. **(A)** Number of annotated genes in the KEGG database on level 1. **(B)** Relative abundances of the six pathways in each sample. Hm, healthy individuals; Pm, patients with periodontitis alone; Cm, patients with Crohn’s disease and periodontitis.

Of the 435,360 predicted genes, 315,285 (72.42%) were annotated in the KEGG database, of which 165,983 (38.13%) were annotated to 4,268 KEGG Ortholog Groups. The numbers of genes involved in carbohydrate metabolism and amino acid metabolism were the largest among the salivary microorganisms, accounting for 10.0% (n = 16,625) and 7.9% (n = 13,071), respectively. Additionally, these genes were mainly involved in cofactor and vitamin metabolism, nucleotide metabolism, membrane transport, energy metabolism, translation, and replication and repair (**Figure 4A**). However, the Hm samples exhibited higher consistency in the relative abundance of these six pathways than did the Pm and Cm samples (**Figure 4B**).

**Figure 5:**
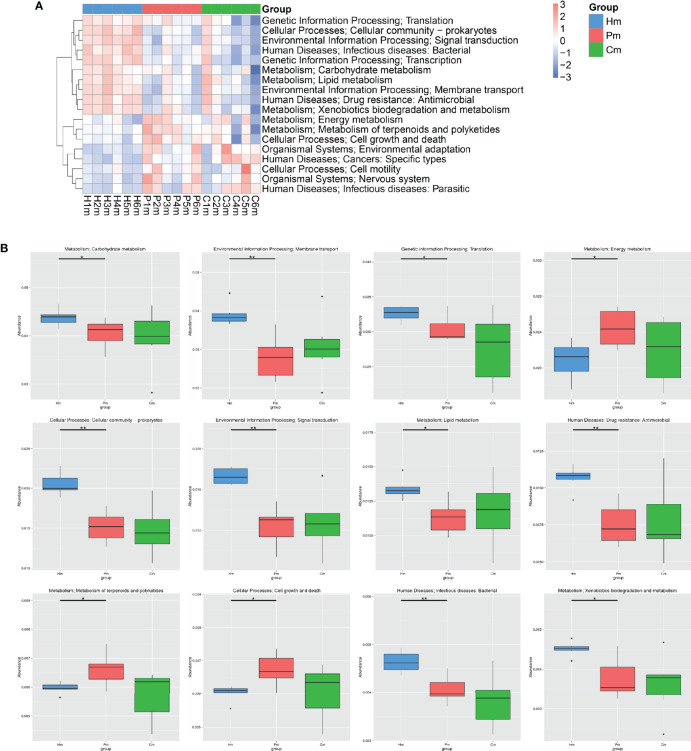
KEGG level 2 pathway annotation according to the genetic data of the oral microbiomes from individuals in the Hm, Cm, and Pm groups. **(A)** Heatmap of the distribution of the top 18 KEGG pathways at the genus level. **(B)** Boxplots indicating the relative abundances of the top 12 KEGG pathways (*q-value < 0.05; **q-value < 0.01; Metastats). Hm, healthy individuals; Pm, patients with periodontitis alone; Cm, patients with Crohn’s disease and periodontitis.

The KEGG level 2 analysis indicated that the top 18 KEGG pathways were those related to translation, the cellular community, signal transduction, infectious diseases, transcription, carbohydrate metabolism, lipid metabolism, membrane transport, drug resistance, xenobiotic biodegradation and metabolism, energy metabolism, cell growth and death, the metabolism of terpenoids and polyketides, environmental adaptation, cancers, cell motility, the nervous system, and infectious diseases (**Figure 5A**). Compared with the Hm group, the Pm group had a lower abundance of pathways for carbohydrate metabolism, membrane transport, translation, cellular community, signal transduction, lipid metabolism, drug resistance, infectious diseases, and xenobiotic biodegradation and metabolism (*p* < 0.05), whereas it was significantly enriched in the pathways for energy metabolism, cell growth and death, and the metabolism of terpenoids and polyketides (*p* < 0.01) (**Figure 5B**). Moreover, there were no significant differences in the abundances of these above-mentioned KEGG pathways between the Cm and Pm groups (**Figure 5B**).

**Figure 6:**
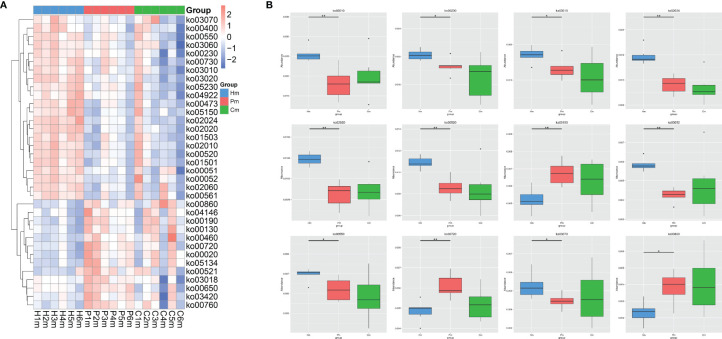
KEGG level 3 pathway annotation according to the genetic data of the oral microbiomes from individuals in the Hm, Cm, and Pm groups. **(A)** Heatmap of the distribution of the top 35 KEGG pathways at the genus level. **(B)** Boxplots indicating the relative abundances of the top 12 KEGG pathways (*q-value < 0.05; **q-value < 0.01; Metastats). Hm, healthy individuals; Pm, patients with periodontitis alone; Cm, patients with Crohn’s disease and periodontitis.

The KEGG level 3 classification analysis revealed 35 pathways that commonly showed differential abundances among the Hm, Pm, and Cm groups (**Figure 6A**). The pathways related to ABC transporters (ko02010), purine metabolism (ko00230), ribosomes (ko03010), quorum sensing (ko02024), the two-component system (ko02020), amino sugar and nucleotide sugar metabolism (ko00520), galactose metabolism (ko00052), peptidoglycan biosynthesis (ko00550), and the bacterial secretion system (ko03070) were blunted in the Pm group. By contrast, the abundance of pathways related to oxidative phosphorylation (ko00190), carbon fixation (ko00720), and porphyrin and chlorophyll metabolism (ko00860) was higher in the Pm group than in the Hm group (**Figure 6B**). There were no significant differences in the abundance of these 12 pathways between the Pm and Cm groups (**Figure 6B**).

### ARO Gene Composition in the Hm, Cm, and Pm Groups

**Figure 7:**
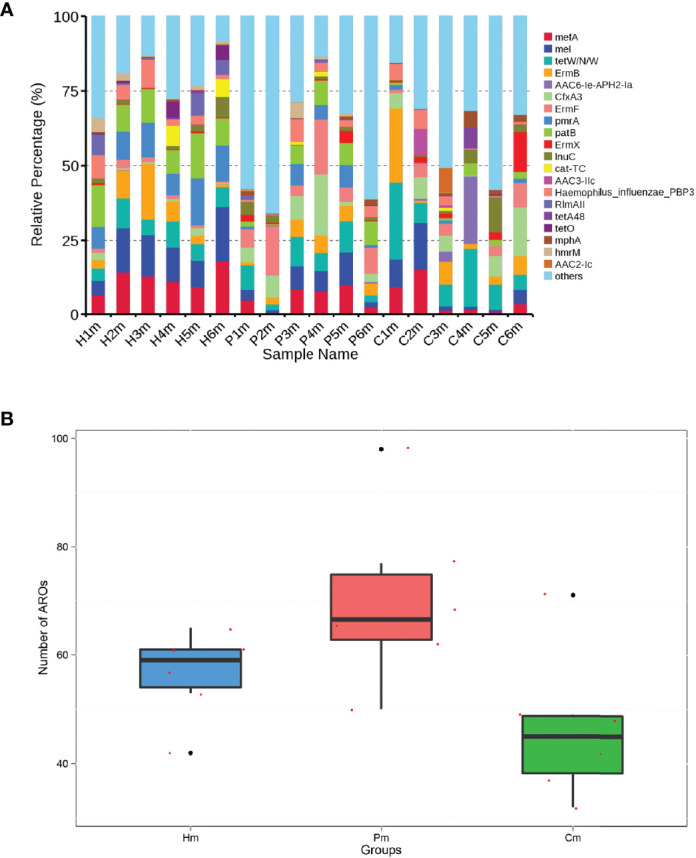
Number of ARO genes and relative abundances of the top 20 ARO genes shared among the individuals in the Pm, Cm, and Hm groups. **(A)** Proportion of the top 20 ARO genes and other ARO genes. **(B)** Differences in the number of ARO genes among the Cm, Pm, and Hm groups. ARO, antibiotic resistance ontology; Hm, healthy individuals; Pm, patients with periodontitis alone; Cm, patients with Crohn’s disease and periodontitis.

After the original deduplication of the gene dataset, 435,360 predicted genes remained. Of these, 175 genes were aligned to the CARD database, including 149 ARO genes. The common top 20 ARO genes identified in the individuals in the Hm, Cm, and Pm groups were *mefA*, *mel*, *tetW/N/W*, *ErmB*, *AAC6-le-APH2-la*, *CfxA3*, *ErmF*, *pmrA*, *patB*, *ErmX*, *InuC*, *cat-TC*, *AAC3-IIc*, *Haemophilusinfluenzae_PBP3*, *RlmAII*, *tetA48*, *tetO*, *mphA*, *hmrM*, and *AAC2-lc* (**Figure 7A**). The relative percentage of *mefA* was higher in the Hm group than in the Pm or Cm groups (**Figure 7A**). Approximately 60 ARO genes were found in the Hm group, 80 in the Pm group, and 50 in the Cm group, indicating that periodontitis and CD could change the composition of these genes (**Figure 7B**).

## Discussion

In this study, a metagenomic analysis was performed on saliva samples from patients with periodontitis alone, patients with both CD and periodontitis, and healthy individuals. It has previously been demonstrated that patients with CD-associated periodontitis have a relatively specific salivary microbiome composition at the phylum and genus levels, which may lead to the execution of relatively unique pathogenic mechanisms in the periodontal tissues (Zhang et al., 2020). However, in that study, the amplification and sequencing of the V_3_–V_4_ region of the microbial 16S rRNA genes did not comprehensively reflect the changes in the oral microecology, and some closely related species were difficult to distinguish from one another. Nonetheless, those authors found a link between salivary microbiota functions and systemic conditions in patients with CD.

A recent meta-analysis revealed a positive association between CD and periodontitis, suggesting that the former increases the prevalence of the latter (She et al., 2020). This finding was supported by another case-control study of age- and sex-matched patients with apical periodontitis and inflammatory bowel disease (Poyato-Borrego et al., 2020). In our study cohort, patients with both CD and periodontitis showed severe periodontal destruction with the highest DMFT scores. The causal relationship between periodontitis and CD can be concluded to be from the interplay between the microbiota and host immune inflammatory response as well as genetic and environmental factors (Figueredo et al., 2011; Zhang et al., 2021). Moreover, researchers have suggested that dysbiosis of the gut microbiota not only advances the development of intestinal disorders but also induces apical periodontitis and periodontal disease (Piras et al., 2017; Poyato-Borrego et al., 2020). It is worth mentioning that our analysis of the phylum-level distribution of the oral microbiota revealed that the composition of the salivary microbiome inferred from the genetic data of the Cm group had changed relative to that of the Pm group. The oral microbiota functions as a community; once the overall composition of the microenvironment changes, pathogenic bacteria cannot be antagonized by beneficial taxa, leading to the development of diseases.

It has been noted in previous studies that the oral microbiota plays a role in the pathogenesis of CD (Xun et al., 2018). Limited knowledge suggests that CD aggravates the dysbiosis of the oral microbiota, which contributes to periodontitis. Therefore, we proposed an association between periodontitis and CD in relation to the oral microbiota, which may be bidirectional. In patients with both CD and periodontitis, the saliva was enriched with *Prevotella nigrescens* and *Prevotella intermedia*, which are reported to be significantly associated with periodontitis (Maeda et al., 1998; Zhang et al., 2017; Zhang et al., 2020). Additionally, the abundance of beneficial bacteria, such as *Streptococcus* and *Gemella*, was lower in the Cm group than in the Pm group. This evidence supports the assumption that dysbiosis of oral microbiota leads to the unfavorable tolerance of patients with CD to periodontal pathogenic bacteria.

The major functions of the oral microbiota include carbohydrate metabolism, amino acid metabolism, vitamin anabolism, energy metabolism, and membrane transport. It has been highlighted that ribosome biogenesis and energy metabolism are exhausted in the active phase of CD (Zhang et al., 2020). Moreover, several studies have shown links between the substantial antibiotic resistance of the human microflora and diverse ARO genes, where a selection of metagenomic functions represented the functional repertoire of those genes (Sommer et al., 2010; Baron et al., 2018; Almeida et al., 2020). Among the ARO genes identified to be harbored by the microbiomes of the individuals in our three study groups, *mefA* occurred predominantly in the Hm group. The presence of *mefA* in *Streptococcus pneumoniae* determines resistance to macrolide (Ubukata et al., 2003). Moreover, periodontitis and CD changed the composition of the ARO genes.

In this study, we found that the pathway related to the two-component system was blunted in the Pm and Cm groups. The bacterial two-component system involved in phosphate uptake in periodontitis induces microbial adaptation to changes in the host environment by regulating gene expression and the maturation and transportation of virulence factors. Short-chain organic acids, including butanoate, have been recognized as important metabolic markers of periodontal inflammation (Ikeda et al., 2020). *Porphyromonas gingivalis* produces butyric acid and isovaleric acid, *Prevotella intermedia* is involved in the generation of propionic acid and butyric acid, and *Fusobacterium nucleatum* also participates in butyric acid metabolism (Huang et al., 2011). In accordance with these findings, we observed that the butyric acid metabolic pathway was enriched in both the Cm and Pm groups. It has been found that organic acid-treated basal cells stop dividing and proliferating, which results in the separation of epithelial cells from the tooth surface at the bound epithelium and contributes to the formation of periodontal pockets (Mackenzie and Gao, 1993). Additionally, butyrate inhibits histone deacetylase by decreasing the expression of cyclin B1, a cell cycle promoter, thereby increasing the acetylation level of histones and thus affecting gene expression (Archer et al., 2005). These results suggest that microbial metabolites can travel from the oral cavity to the entire body and affect host health.

Previous studies had proposed the changes on salivary microbial composition of periodontitis patients or of CD patients. However, these precious studies were performed based on 6S rRNA gene sequencing. In addition, less study focused on the associations of CD and periodontitis. Our current study characterized the salivary microbiota in patients with CD-related periodontitis and periodontitis based on a metagenomic analysis, filling up the vacancy. However, there were also limitations. The limited number of patients (only six samples each group) was the major limiation of this study. Although there were also studies performed based on samll sample size (Zeng et al., 2019; Dos Santos et al., 2020), we advocate that convincing conclusions can be proposed based on only large number of samples. In addition, potential influencing factors such as diet, lifestyle factors (such as socio-economic status and the attention to oral hygiene) that may influence oral microbiota composition were not considered, which may be confounding factors to the results. Therefore, further investigations should be performed based on large sample size after excluding influencing factors.

## Conclusions

In summary, we revealed significant structural changes in the salivary microbiota at the species level in patients with CD and periodontitis. A significant decrease in the abundance of beneficial bacteria (e.g., *Streptococcus mitis*) and an increase in periodontal pathogens (e.g., *Prevotella nigrescens* and *Prevotella intermedia*) will lead to poorer tolerance to periodontal pathogenic bacteria and greater susceptibility to concomitant periodontitis in patients with CD. Our findings suggest that salivary metabolic pathways are significantly altered in periodontitis and CD, and the downregulation of some metabolic pathways (e.g., for carbohydrates and butyric acid) affects local metabolism in the oral cavity, which in turn has potential effects on the oral microecology in patients with CD.

## Data Availability Statement

The raw sequencing data were deposited in the National Center for Biotechnology Information. (NCBI) Sequence Read Archive (SRA) database under accession number PRJNA741688.

## Author Contributions

BS and TG conceived of and designed the study. BL participated in the acquisition of data. XG analyzed and interpreted the data. KX and LX participated in the design of the study and performed the statistical analysis. BS and TG helped draft the manuscript and revise it for important intellectual content. All authors contributed to the article and approved the submitted version.

## Funding

This work was supported by the Social Development Foundation of Jiangsu Province (Program No. BE2019623) and the Foundation of Jiangsu Commission of Health (Program Nos. H2018043, LGY2019010, and QNRC2016906).

## Conflict of Interest

The authors declare that the research was conducted in the absence of any commercial or financial relationships that could be construed as a potential conflict of interest.

## Publisher’s Note

All claims expressed in this article are solely those of the authors and do not necessarily represent those of their affiliated organizations, or those of the publisher, the editors and the reviewers. Any product that may be evaluated in this article, or claim that may be made by its manufacturer, is not guaranteed or endorsed by the publisher.
