# Supplementary material for: Metagenomic Analysis of Saliva Reveals Disease-Associated Microbiotas in Patients With Periodontitis and Crohn’s Disease-Associated Periodontitis
Source: Front Cell Infect Microbiol. 2021 Sep 27;11:719411. doi: 10.3389/fcimb.2021.719411 (PMC8504578; doi:10.3389/fcimb.2021.719411)
Supplement: Supplementary file 1 [file Table_1.docx]

Supplementary Material

Metagenomic Analysis of Saliva Reveals Disease-associated Microbiotas in Patients with Periodontitis and Crohn’s Disease-related Periodontitis

Boyang Sun, Bingyao Liu, Xiaojiao Gao, Kai Xing, Li Xie, Ting Guo


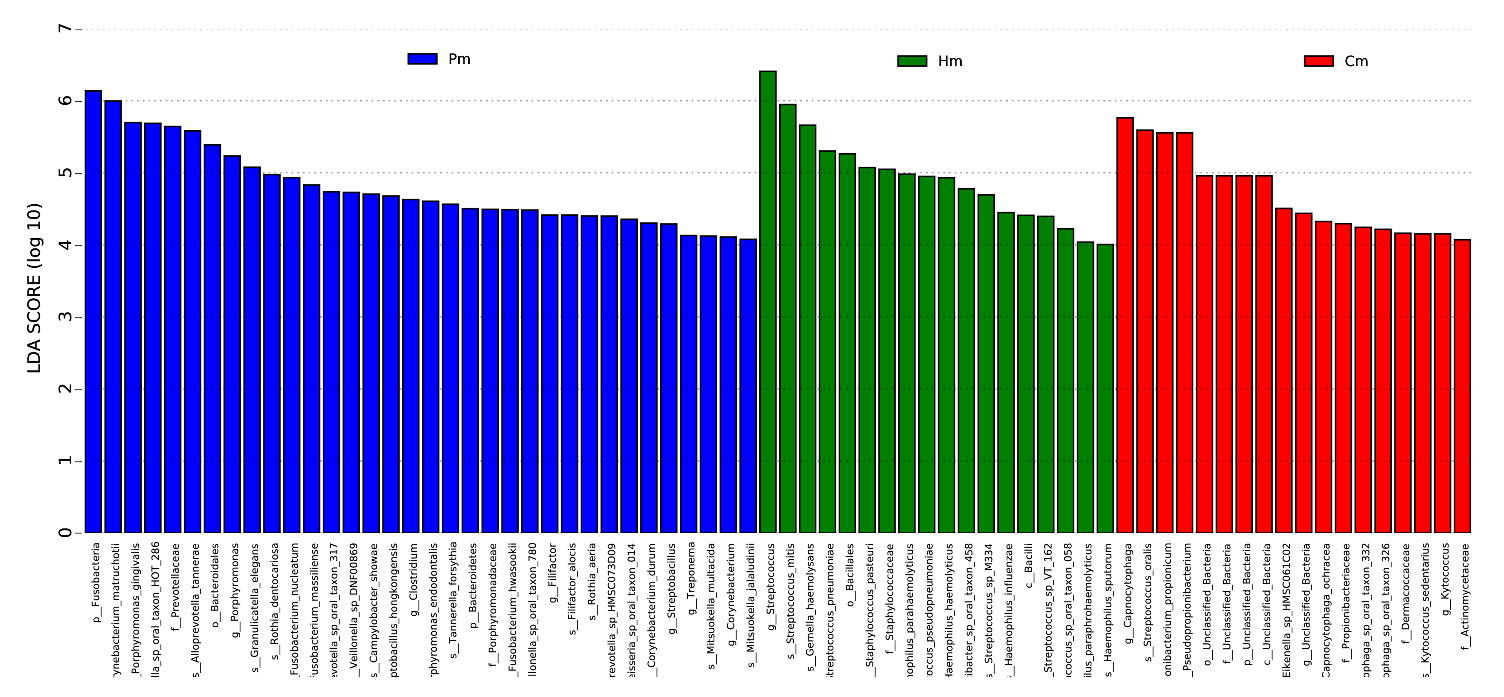


**Supplementary Figure S1.** LEfSe analysis of the differentially abundant taxa in the microbiotas of the three study groups. LDA scores based on the LEfSe analysis are displayed. LEfSe, linear discriminant analysis effect size; LDA, linear discriminant analysis; Hm, healthy individuals; Pm, patients with periodontitis alone; Cm, patients with Crohn’s disease and periodontitis.

**Table S1.** Statistics of the sequencing data.

| Sample | Insert size (bp) | Seq Strategy | Raw data | Raw reads | Clean data | Clean_Q20 | Clean_Q30 | Clean_GC (%) | Effective (%) | Non-host data |
| --- | --- | --- | --- | --- | --- | --- | --- | --- | --- | --- |
| C1m | 350 | (150:150) | 10,117.28 | 67,448,508 | 10,109.18 | 97.69 | 93.41 | 41.03 | 99.92 | 1,240.94 |
| C2m | 350 | (150:150) | 10,097.89 | 67,319,280 | 10,090.67 | 97.4 | 92.77 | 41.61 | 99.928 | 920.3 |
| C3m | 350 | (150:150) | 10,332.88 | 68,885,874 | 10,324.53 | 97.27 | 92.56 | 42.27 | 99.919 | 1,238.68 |
| C4m | 350 | (150:150) | 10,844.25 | 72,295,012 | 10,835.30 | 97.27 | 92.5 | 41.09 | 99.917 | 264.12 |
| C5m | 350 | (150:150) | 10,429.36 | 69,529,038 | 10,422.26 | 97.31 | 92.59 | 41.23 | 99.932 | 521.74 |
| C6m | 350 | (150:150) | 10,267.15 | 68,447,698 | 10,257.26 | 97.22 | 92.4 | 41.09 | 99.904 | 339.96 |
| H1m | 350 | (150:150) | 10,277.30 | 68,515,364 | 10,267.83 | 97.23 | 92.39 | 40.89 | 99.908 | 964.16 |
| H2m | 350 | (150:150) | 10,154.25 | 67,694,992 | 10,145.76 | 97.36 | 92.64 | 40.73 | 99.916 | 1,395.36 |
| H3m | 350 | (150:150) | 10,267.74 | 68,451,630 | 10,259.60 | 97.19 | 92.27 | 40.86 | 99.921 | 1,873.78 |
| H4m | 350 | (150:150) | 10,540.92 | 70,272,792 | 10,532.04 | 97.38 | 92.72 | 41.24 | 99.916 | 1,107.82 |
| H5m | 350 | (150:150) | 10,023.57 | 66,823,822 | 10,016.80 | 97.22 | 92.37 | 40.88 | 99.932 | 974.5 |
| H6m | 350 | (150:150) | 10,141.88 | 67,612,526 | 10,134.63 | 97.27 | 92.49 | 40.42 | 99.929 | 587.84 |
| P1m | 350 | (150:150) | 10,518.11 | 70,120,706 | 10,509.92 | 97.3 | 92.54 | 41.48 | 99.922 | 790.44 |
| P2m | 350 | (150:150) | 17,879.17 | 119,194,488 | 17,869.63 | 97.31 | 92.5 | 43.54 | 99.947 | 6,793.80 |
| P3m | 350 | (150:150) | 10,186.74 | 67,911,610 | 10,178.49 | 97.57 | 93.06 | 41.34 | 99.919 | 3,144.18 |
| P4m | 350 | (150:150) | 10,660.63 | 71,070,850 | 10,653.71 | 97.64 | 93.18 | 41.88 | 99.935 | 4,367.38 |
| P5m | 350 | (150:150) | 10,139.71 | 67,598,044 | 10,129.29 | 97.47 | 92.89 | 41.57 | 99.897 | 1,055.04 |
| P6m | 350 | (150:150) | 11,091.76 | 73,945,048 | 11,083.36 | 97.33 | 92.63 | 41.31 | 99.924 | 472.18 |
| Total raw data: 193,970.59 Mbp | | | | | | | | | | |
| Average raw data: 10,776.14 Mbp | | | | | | | | | | |
| Total clean data: 193,820.26 Mbp | | | | | | | | | | |
| Average clean data: 10,767.79 Mbp | | | | | | | | | | |
| Effective percentage: 99.92% | | | | | | | | | | |
| Total non-host data: 28,052.22 Mbp | | | | | | | | | | |
| Average non-host data: 1,558.46 Mbp | | | | | | | | | | |
| Effective rate: 14.47% | | | | | | | | | | |

**Table S2.** Statistics of the predicted genes.

| **Sample** | **No. of ORFs** | **Integrity: none** | **Integrity: end** | **Integrity: start** | **Complete ORFs (percentage)** | **Total length (Mbp)** | **Average length (bp)** | **GC percentage** |
| --- | --- | --- | --- | --- | --- | --- | --- | --- |
| C1m | 27,651 | 1,784 (6.45%) | 5,151 (18.63%) | 5,667 (20.49%) | 15,049 (54.42%) | 18.69 | 675.84 | 43.14 |
| C2m | 26,846 | 2,002 (7.46%) | 4,739 (17.65%) | 5,483 (20.42%) | 14,622 (54.47%) | 19.07 | 710.44 | 48.61 |
| C3m | 60,928 | 4,826 (7.92%) | 12,481 (20.48%) | 14,839 (24.35%) | 28,782 (47.24%) | 38.8 | 636.77 | 50.29 |
| C4m | 8,719 | 560 (6.42%) | 1,883 (21.6%) | 1,844 (21.15%) | 4,432 (50.83%) | 5.08 | 582.21 | 42.15 |
| C5m | 30,342 | 4,559 (15.03%) | 8,243 (27.17%) | 10,590 (34.9%) | 6,950 (22.91%) | 15.7 | 517.42 | 51.6 |
| C6m | 8,570 | 1,170 (13.65%) | 2,374 (27.7%) | 2,719 (31.73%) | 2,307 (26.92%) | 3.81 | 444.71 | 52.73 |
| H1m | 36,076 | 3,819 (10.59%) | 8,937 (24.77%) | 10,214 (28.31%) | 13,106 (36.33%) | 21.64 | 599.77 | 41.7 |
| H2m | 47,382 | 5,388 (11.37%) | 11,715 (24.72%) | 13,785 (29.09%) | 16,494 (34.81%) | 28.16 | 594.23 | 40.93 |
| H3m | 42,636 | 4,481 (10.51%) | 10,305 (24.17%) | 11,623 (27.26%) | 16,227 (38.06%) | 26.71 | 626.37 | 43.31 |
| H4m | 33,437 | 3,133 (9.37%) | 7,543 (22.56%) | 8,956 (26.78%) | 13,805 (41.29%) | 20.9 | 625.18 | 48.72 |
| H5m | 30,782 | 3,913 (12.71%) | 8,495 (27.6%) | 9,722 (31.58%) | 8,652 (28.11%) | 16.99 | 551.81 | 39.56 |
| H6m | 15,338 | 828 (5.4%) | 3,034 (19.78%) | 2,832 (18.46%) | 8,644 (56.36%) | 10.55 | 687.57 | 37.89 |
| P1m | 18,244 | 2,181 (11.95%) | 4,550 (24.94%) | 5,531 (30.32%) | 5,982 (32.79%) | 10.16 | 556.91 | 52.57 |
| P2m | 213,058 | 23,484 (11.02%) | 51,790 (24.31%) | 62,459 (29.32%) | 75,325 (35.35%) | 129.91 | 609.75 | 47.65 |
| P3m | 86,715 | 8,808 (10.16%) | 19,582 (22.58%) | 22,241 (25.65%) | 36,084 (41.61%) | 57.22 | 659.92 | 44.25 |
| P4m | 84,775 | 8,339 (9.84%) | 18,474 (21.79%) | 21,360 (25.2%) | 36,602 (43.18%) | 56.35 | 664.75 | 42.35 |
| P5m | 38,968 | 5,865 (15.05%) | 10,390 (26.66%) | 13,156 (33.76%) | 9,557 (24.53%) | 20.34 | 522.08 | 46.54 |
| P6m | 6,812 | 490 (7.19%) | 1,745 (25.62%) | 1,716 (25.19%) | 2,861 (42%) | 3.27 | 480.07 | 46.45 |
| Total open reading frames (ORFs): 817,279 | | | | | | | | |
| Average ORFs: 45,404 | | | | | | | | |
| No. of ORFs: 435,360 | | | | | | | | |
| Integrity: none: 39,180 (9%) | | | | | | | | |
| Integrity: end: 91,461 (21.01%) | | | | | | | | |
| Integrity: start: 104,358 (23.97%) | | | | | | | | |
| Complete ORF number: 200,361 | | | | | | | | |
| Complete ORF percentage: 200,361 (46.02%) | | | | | | | | |
| Total length (Mbp): 284.96 | | | | | | | | |
| Average length (bp): 654.54 | | | | | | | | |
| GC percentage: 45.95 | | | | | | | | |
